# A randomized phase II trial on the addition of dutasteride to combined androgen blockade therapy versus combined androgen blockade therapy alone in patients with advanced or metastatic salivary duct carcinoma – the DUCT study protocol

**DOI:** 10.1186/s12885-024-12889-0

**Published:** 2024-09-20

**Authors:** Jetty A. M. Weijers, Gerald W. Verhaegh, G. Lassche, Adriana C. H. van Engen-van Grunsven, Chantal M. L. Driessen, Nielka P. van Erp, Marianne A. Jonker, Jack A. Schalken, Carla M. L. van Herpen

**Affiliations:** 1 Department of Medical Oncology, Radboud Institute for Medical Innovation, Radboud University Medical Center, Geert Grooteplein Zuid 8, Nijmegen, 6525 GA The Netherlands; 2 Department of Urology, Radboud Institute for Medical Innovation, Radboud University Medical Center, Geert Grooteplein Zuid 8, 6525 GA Nijmegen, The Netherlands; 3 Department of Pathology, Radboud Institute for Medical Innovation, Radboud University Medical Center, Geert Grooteplein Zuid 8, 6525 GA Nijmegen, The Netherlands; 4 Department of Pharmacy, Radboud Institute for Medical Innovation, Radboud University Medical Center, Geert Grooteplein Zuid 8, 6525 GA Nijmegen, The Netherlands; 5 Science Department IQ Health, Radboud Institute for Medical Innovation, Radboud University Medical Center, Geert Grooteplein Zuid 8, 6525 GA Nijmegen, The Netherlands

**Keywords:** Rare Cancer, Salivary Duct Carcinoma, Androgen Receptor, Combined Androgen Blockade, Androgen Deprivation Therapy, Dutasteride, Steroid 5α-reductase, Anti-hormonal therapy, Systemic therapy, Salivary gland cancer

## Abstract

**Background:**

Salivary duct carcinoma (SDC) is a rare and aggressive subtype of salivary gland cancer, frequently associated with incurable recurrences and distant metastases (R/M). Proliferation of SDC relies on androgen receptor (AR) signalling, prompting the use of combined androgen blockade (CAB, *i.e.*, luteinizing hormone-releasing hormone agonist and/or AR antagonists) to R/M SDC patients. However, only a subset of patients benefits from such treatments. We have shown that response to CAB is associated with *steroid 5α-reductase 1* (*SRD5A1*) mRNA expression. SRD5A1 catalyses the intracellular conversion of testosterone into the more potent AR-agonist dihydrotestosterone. This conversion can be inhibited by dutasteride, a potent SRD5A1-inhibitor, which is currently prescribed for benign prostatic hyperplasia. We hypothesize that repurposing dutasteride to target AR signalling in SDC could enhance therapeutic response and clinical outcome in SDC patients.

**Methods:**

This prospective, open-label, randomized controlled phase II clinical trial, is designed to investigate whether dutasteride as an adjunct drug to CAB improves response rate and clinical outcome in patients with AR-positive R/M SDC. Patients are divided in two cohorts based on their prior systemic treatments. In cohort A, CAB-naïve patients (*n* = 74) will be randomly assigned to either a control arm (Arm 1) receiving CAB (goserelin 10.8 mg/3m and bicalutamide 50 mg/OD) or an experimental arm (Arm 2) where dutasteride (0.5 mg/OD) is added to the CAB regimen. In cohort B, patients with disease progression after adjuvant or first-line palliative CAB therapy (max. *n* = 24) will receive goserelin, bicalutamide, and dutasteride to assess whether the addition of dutasteride can overcome therapy resistance. The primary endpoints are the objective response rate and duration of response. Secondary endpoints are progression-free survival, overall survival, clinical benefit rate, quality of life, and safety. Translational research will be performed to explore molecular target expression differences and their correlation with clinical outcome.

**Discussion:**

The DUCT study addresses an unmet medical need by investigating the repurposing of dutasteride to enhance treatment response and improve clinical outcome for patients with R/M SDC, especially those with limited alternative treatment options, such as HER2-negative cases. By repurposing a registered low-cost drug, this trial’s findings could be readily applied into clinical practice.

**Trial registration:**

Clinicaltrials.gov Identifier: NCT05513365. Date of registration: August 24, 2022.

**Protocol version:**

Current protocol version 4.0, February 21, 2024.

## Background

Salivary gland cancer (SGC) is a rare cancer, exhibiting an annual incidence of approximately 1 to 2 new cases per 100,000 individuals [1, 2]. The World Health Organization (WHO) classifies over twenty malignant epithelial salivary gland tumour types based on histological and clinical features [3]. Salivary duct carcinoma (SDC), represents one of the most aggressive subtypes, predominantly affecting the parotid gland [2]. In SDC, incurable local recurrences and/or distant metastases (R/M) are common and treatment options are limited, especially when human epidermal growth factor 2 (HER2) status is negative or when no other druggable genetic alterations are present in the tumour [3–5]. Half of all patients already present with locoregional or distant metastases at diagnosis or will develop metastases throughout the course of their disease [6]. Distant metastases are predominantly present in the lungs (54% of patients with distant disease), bones (46%), and brain (18%) [2]. The median overall survival (OS) in R/M SDC is only 5 months when best supportive care is given [7].

SDC, like prostate cancer (PCa), relies on the androgen receptor (AR) signalling pathway for tumour growth, and therefore androgen-receptor signalling inhibitors (ARSI) and androgen deprivation therapy (ADT) have been studied [2, 8]. In a phase II trial, the efficacy of combined androgen blockade (CAB), consisting of a luteinizing hormone-releasing hormone (LHRH) agonist (*i.e.,* leuprorelin acetate 3.75 mg/4w) in combination with an anti-androgen (*i.e.*, bicalutamide 80 mg/OD) has been studied in advanced SDC patients. The objective response rate (ORR) was 42% (11% complete response (CR) and 31% partial response (PR)), median progression free survival (PFS) and OS were 8.8 months and 30.5 months, respectively [4]. Based on this trial and retrospective case series, the ASCO guidelines [9] and the ESMO guidelines [6] strongly recommend to treat AR-positive R/M SDC patients with CAB. Still, there remains an unmet clinical need for improvement of current anti-hormonal treatments and/or novel systemic treatment strategies, but the rarity of SDC hampers the performance of large clinical trials.

Previous translational research has demonstrated that high levels of *SRD5A1* mRNA, encoding for the 5-alpha reductase type 1 enzyme, is predictive for clinical benefit and prolonged PFS and OS in CAB-treated AR-positive SDC patients [10]. SRD5A1 catalyses the intracellular conversion of testosterone into the more potent AR agonist dihydrotestosterone. Based on this mechanism, it is hypothesized that patients with high *SRD5A1* levels are highly dependent on androgens for their survival, and hence, benefit more from AR-targeting therapies. Notably, both isoforms of 5-alpha reductase, type I and type II, are inhibited by dutasteride, an oral anti-androgenic compound. In clinical practice, dutasteride has been approved for the treatment of benign prostate hyperplasia, a prevalent condition in elderly men. The extensive experience with prolonged use of dutasteride has demonstrated a relatively-well tolerability with a low incidence of (severe) side-effects [11]. In addition to the current approved indication, the efficacy of dutasteride has been investigated in several PCa trials. In a randomized placebo-controlled phase II trial, the efficacy of dutasteride monotherapy was evaluated in 294 patients with advanced PCa. Dutasteride significantly delayed the time to prostate-specific antigen (PSA) doubling compared with placebo after 2 years of treatment, along with suppressed tumour growth demonstrating an overall relative risk reduction of 59% in favour of dutasteride compared to placebo (*p* < *0.001*) [12]. The combination of a 5-alpha reductase inhibitor and CAB has also been studied in PCa, indicating that dutasteride in combination with anti-androgens could be of added value in localized or even locally advanced PCa [13–16]. For example, in a randomized clinical trial, 59 patients with localized PCa have been treated with an LHRH agonist and an anti-androgen with or without the 5-alpha reductase inhibitor finasteride. Notably, patients treated with CAB plus finasteride exhibited a significantly longer median time to relapse based on their PSA increase (34 months) compared to patients receiving CAB alone (19 months; *p* = *0.013*) [14, 15]. Recently, dutasteride has been investigated as a second-line therapy in combination with abiraterone in two patients with abiraterone-resistant PCa. Although limited to a sample size of two, the results suggests that dutasteride may improve the clinical efficacy of abiraterone in abiraterone-resistant PCa [17]. Furthermore, an in vitro study demonstrated a synergistic inhibition of prostate tumour cell proliferation by combining a 5-alpha reductase inhibitor and an AR antagonist [18].

**Fig. 1:**
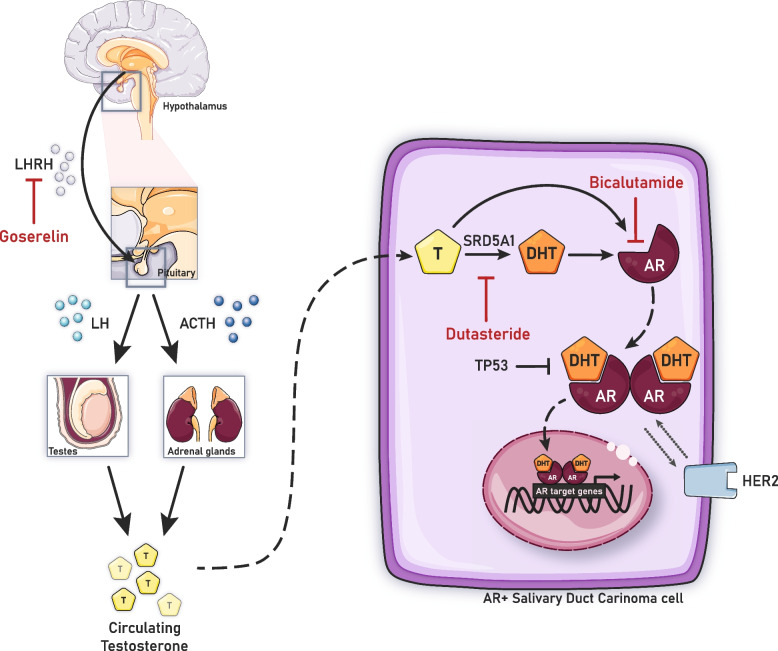
Simplified version of the androgen receptor pathway in salivary duct carcinoma, including the androgen-receptor signalling inhibitors under investigation in the DUCT study. The uncertain interaction between the HER2 receptor and AR is represented by the dotted grey arrows. Abbreviations: ACTH, adrenocorticotropic hormone; AR, androgen receptor; DHT, dihydrotestosterone; HER2, human epidermal growth factor 2; LH, luteinizing hormone; LHRH, luteinizing hormone-releasing hormone; SDC, salivary duct carcinoma; SRD5A1, 5α-reductase 1; TP53, tumour protein p53

Based on the overexpression of *SRD5A1* in CAB-responsive R/M SDC, and the efficacy of dutasteride in several trials involving men with prostate cancer, dutasteride could also be a valuable addition to the armamentarium against SDC. Hence, these findings provide a rationale for considering dutasteride as an adjunct drug to CAB therapy, offering a promising novel treatment approach to improve therapy response and clinical outcomes in patients with this aggressive rare cancer (Fig. 1). To date, no clinical trials have been conducted to assess the efficacy and safety of dutasteride as an adjunct drug in SDC patients undergoing anti-hormonal therapy. Here, we detail a prospective, open-label, randomized controlled phase II clinical trial, designed to investigate whether dutasteride as an adjunct drug to CAB improves therapy response and clinical outcome in patients with AR-positive R/M SDC.

## Objectives

The primary objectives of the DUCT study are to evaluate the ORR of dutasteride in combination with CAB patients with R/M SDC together with the duration of response (DoR), and to evaluate whether addition of dutasteride counteracts therapy resistance in patients who previously have been treated with anti-hormonal therapy. The ORR is defined as the proportion of participants who have CR or PR determined per RECIST v1.1. The DoR is defined as the time from first tumour assessment at which the objective response was recorded as CR or PR that is subsequently confirmed until PD determined per RECIST v1.1, or death from any cause, whichever occurs first.

The secondary objectives are:

- To assess the PFS, defined as the time from trial enrolment until date of first documented disease progression per RECIST v.1.1, or death due to any cause, whichever occurs first.
- To assess the OS, defined as the time from trial enrolment to the date of death due to any cause.
- To assess the clinical benefit rate (CBR), including confirmed CR or PR at any time or stable disease (SD) of at least 6 months determined per RECIST v.1.1.
- To assess the quality of life (QoL) of patients treated with anti-hormonal therapy, according to approved EORTC (QLQ-C30, QLQ-H&N43, QLQ-SHQ22) and VAS questionnaires.
- To assess the safety profile of dutasteride combined to CAB therapy by the incidence of severe adverse events (SAE) according to National Cancer institute Common Terminology Criteria for Adverse Events (NCI-CTCAE) version 5.0.
- To explore expression of molecular markers (*e.g., AR*, *SRD5A1* and *SRD5A2*) in patients’ blood and tumour samples to monitor treatment efficacy.

## Methods

### Trial design

The DUCT study is a prospective, open-label, randomized controlled, single-institution (Radboud university medical center; Radboudumc, Nijmegen, The Netherlands), phase II clinical trial, to investigate the efficacy of dutasteride as an adjunct drug to CAB in AR-positive R/M SDC patients. The trial is approved by the Medical research Ethics Committees United (MEC-U) as of August 11, 2022 by Clinical Trials Information System (CTIS) portal [reference EU CT-number: 2022–500745–24–00] and is registered on ClinicalTrials.gov [NCT05513365; date of registration August 24, 2022]. This protocol adheres to the Standard Protocol Items: Recommendations for Interventional Trials (SPIRIT) guidelines [19].

**Fig. 2:**
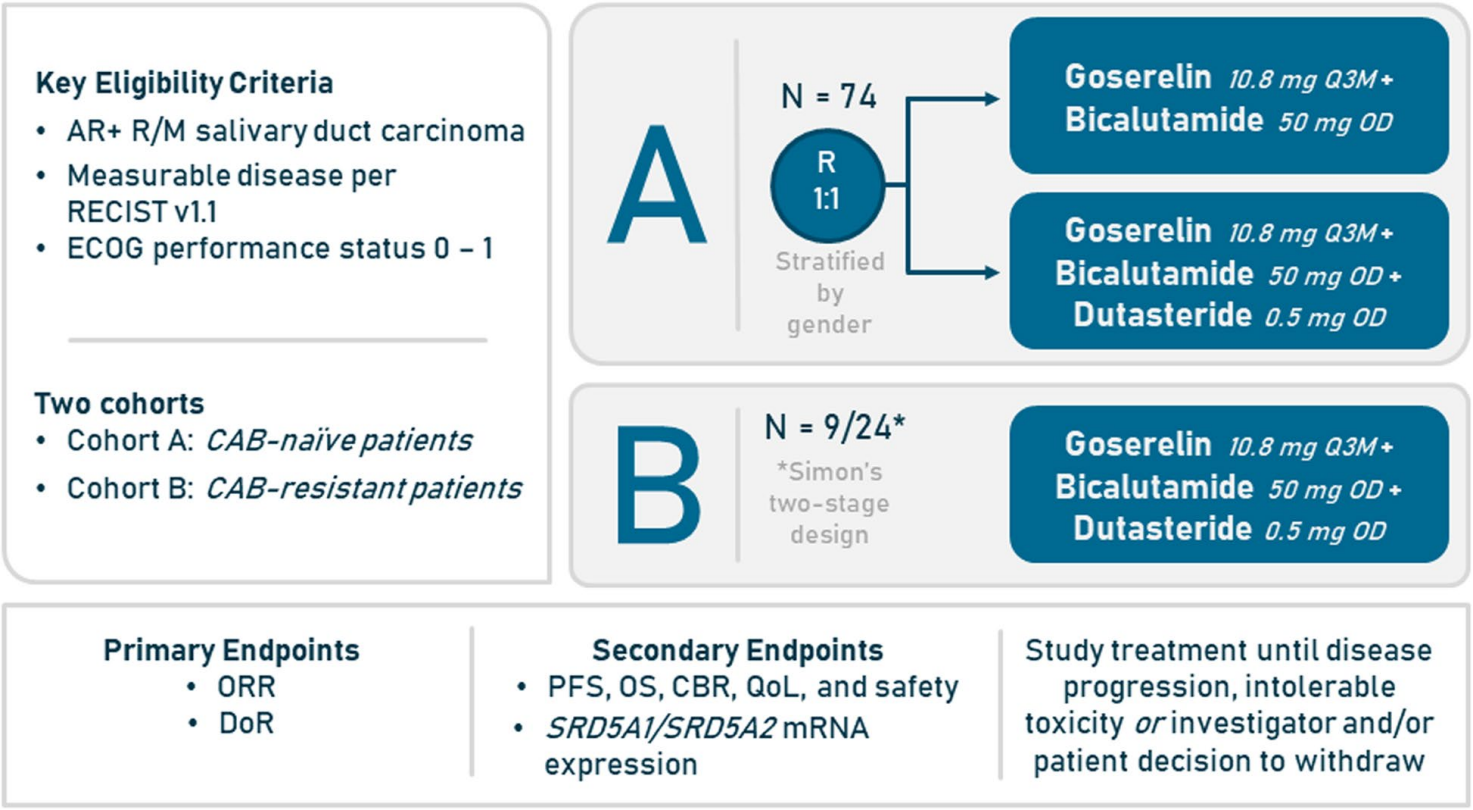
DUCT Study design and enrolment. Abbreviations: AR, Androgen receptor; CAB, Combined androgen blockade; CBR, Clinical benefit rate; DoR, Duration of Response; ECOG, Eastern Cooperative Oncology Group; OD, Once daily; ORR, Objective response rate; OS, Overall survival; PFS, Progression-free survival; QoL, Quality of Life; R/M, Recurrent and/or metastatic

The DUCT study is divided in two cohorts according to prior systemic treatment(s) (Fig. 2). In cohort A, CAB-naïve patients (*n* = 74) will be randomized in a 1:1 ratio, in which the control arm will receive goserelin (10.8 mg/3m) and bicalutamide (50 mg/OD), and the experimental arm will receive goserelin (10.8 mg/3m), bicalutamide (50 mg/OD), and dutasteride (0.5 mg/OD). In cohort B, CAB-resistant patients (*n* = max. 24; *e.g.*, in the adjuvant setting with anti-androgens and/or LHRH-analogues or in the palliative R/M setting progressive on first-line therapy) will be enrolled receiving similar therapy to the experimental arm, *i.e.*, goserelin, bicalutamide, and dutasteride.

### Eligibility criteria

To be eligible for enrolment, subjects must meet the following inclusion criteria: capable of and willing to prove written informed consent; having a confirmed pathologically/histologically diagnosis of AR-positive R/M SDC; measurable disease per RECIST v1.1; age of 18 years or older; Eastern Cooperative Oncology Group (ECOG) performance status of 0 or 1 [20]; and demonstrating adequate bone marrow, liver, renal, and cardiac functions.

Exclusion criteria for trial participation encompass: recent use (< 6 months) of 5-alpha reductase inhibitors; prior allergic reactions to compounds such as goserelin, bicalutamide and/or dutasteride as well as to peanut and/or soy; inadequate swallowing capacity; long QT-syndrome; failure to implement adequate contraceptive measures for patients with reproductive potential; pregnancy or lactation; uncontrolled cardiovascular disorders or hypertension, recent stroke, or myocardial infarction, and serious active infections; any other concurrent experimental treatments; curative radiation therapy within 4 weeks before inclusion; or palliative radiation therapy 1 week before inclusion.

### Facultative biopsies

In the context of translational research, pre-treatment and/or post-treatment facultative biopsies will be obtained. For these biopsies, a separate informed consent is required. The main inclusion criteria for both facultative biopsies is the presence of a safely accessible disease localization of SDC, preferably metastatic disease.

### Dose modifications

In general, dose modifications are recommended for events that, if persistent, could become serious or intolerable, according to the Dutch Medicines Evaluation Board (CBG MEB). No dosage adjustments are required for participants with renal impairment using goserelin, bicalutamide, or dutasteride. Additionally, dutasteride should be used with caution in patients with mild to moderate hepatic impairment due to limited available information and is contraindicated in patients with severe hepatic impairment.

### Trial assessments

Patients will undergo treatment and response evaluations until progressive disease (PD), intolerable toxicity, or investigator and/or patient decision to withdraw. They will attend follow-up visits every three months in accordance with the standard of care for R/M SDC in the Radboudumc, which includes physical examination, blood analysis, and imaging via neck-, chest- and abdominal computed tomography (CT)-scans. Additional blood samples will be collected for translational research purposes. Additionally, eligible patients will be requested to complete multiple QoL questionnaires online using the electronic data management platform *Castor EDC (*http://www.castoredc.com*).* This includes three EORTC QoL questionnaires: the EORTC QLQ-C30 to assess the QoL of the cancer patients, the EORTC QLQ-H&N43 module designed for evaluation of head and neck cancer patients specifically, and the EORTC QLQ-SHQ22 to examine sexual health in patients undergoing anti-hormonal therapy [21–23]. The Visual Analogue Scale (VAS) will be incorporated to assess the presence of pain [24, 25]. Details regarding the complete assessment overview and laboratory parameters are provided in Tables 1 and 2, respectively.

**Table 1:** Schematic overview of trial assessments at specific time points during the DUCT study

	**Time point in clinical trial**				
**Assessment**	**Screening / Baseline**	**Week 12**	**Week 24**	**Every 12 weeks until PD**	**PD**
Informed consent	X				
Eligibility assessment	X				
Physical examination + vital signs	X	X	X	X	
Electrocardiogram	X	X			
Neck-, chest- and abdominal CT-scan	X	X	X	X	
Clinical laboratory tests					
* Haematology parameters*	X	X	X	X	X
* Biochemistry parameters*	X^a^	X	X	X	X
* Thyroid function*	X				
* ctDNA*	X	X	X		X
Quality of life questionnaires					
* EORTC QLQ-C30* * EORTC QLQ-H&N43* * EORTC QLQ-SHQ22* * VAS*	X	X	X	X	X
Toxicity scoring (NCI CTCAE v.5.0)	X	X	X	X	
Facultative tumour biopsy					
* Coagulation parameters*	X^b^				X^b^

Abbreviations: *CT* Computed-tomography, *ctDNA* circulating tumour DNA, *PD* Progressive disease

^a^Including pregnancy test in pre-menopausal female participants

^b^This procedure contains a separate informed consent. Coagulation parameters are only measured in case of pre- and post-treatment facultative tumour biopsies

**Table 2:** Schematic overview of laboratory parameters at specific time points during the DUCT study

		**Time point in clinical trial**				
**Category**	**Parameter**	**Screening / Baseline**	**Week 12**	**Week 24**	**Every 12 weeks from week 24**	**PD**
Haematology	Hemocytometry Automated differential	X	X	X	X	X
Biochemistry	Albumin Alkaline phosphatase ALT AST Bilirubin (direct) Bilirubin (total) Calcium Chloride CRP Gamma-glutamyl transferase LDH Magnesium Phosphate Potassium Sodium Urea	X	X	X	X	X
Kidney function	CKD-EPI-eGFR Creatinine	X	X	X	X	X
Hormones	Testosterone	X	X	X	X	X
Thyroid	Free thyroxine TSH	X				
Pregnancy	HCG blood	X^a^				
Coagulation	APTT INR	X^b^				X^b^
Research	ctDNA	X	X	X		X

Abbreviations: *ALT* Alanine aminotransferase, *AST* Aspartate aminotransferase, *LDH* Lactate dehydrogenase, *CRP* C-reactive protein,* CKD-EPI* Chronic kidney disease epidemiology collaboration, *PD* Progressive disease, *TSH* Thyroid stimulating hormone, HCG Human chorionic gonadotropin, *APTT* Activated partial thromboplastin time, *INR* International normalized ratio, *ctDNA* circulating tumour DNA

^a^In case of premenopausal female patients

^b^In case of pre-treatment and/or post-treatment biopsy

### Follow-up and discontinuation

Follow-up will continue for two years after the last inclusion, or until patient has been deceased, whichever occurs first. Patients can withdraw from the trial at any time without consequences. Once a participant withdraws, no further data or samples will be collected. However, participants who received at least one dose of study treatment, will undergo study-related medical record screening during follow-up independent of withdrawal. If a participant withdraws before start of study medication, there will be no study-related follow-up.

### Translational research

Goal of the translational part of this trial is to identify biomarkers with predictive potential, *i.e.*, markers that can predict response to CAB +/- dutasteride. Expression of relevant/potential biomarkers for resistance mechanisms of CAB will be determined at the transcriptome level. To perform the translational research part of this trial, different patient samples will be used, including blood (serum) and (fresh) tumour tissue. These patients’ samples will be stored in the salivary gland cancer biobank ‘Speekselklierkanker,’ which was previously approved by Committee on Research Involving Human Subjects (CMO) Radboudumc and up and running since 2017 (dossier number: 2017–3697). All samples will be managed in accordance with the biobank standard operating procedures of the Radboudumc.

From the (fresh) pre- and/or post-treatment tumour samples, total RNA will be extracted. These RNA samples will be used to measure the expression of genes involved in or regulated by AR signalling, or genes which affect AR signalling or resistance to CAB. The selection of genes will be based on prostate cancer research, and will, amongst others, include *AKR1C3, AR*, *AR-V7, CYP17A1, KLK3, SRD5A1, SRD5A2,* and *NAALADL2-AS2*. The gene expression profiles will initially be obtained using reverse transcription-quantitative polymerase chain reaction (RT-qPCR) technology, and a full RNA sequencing-based transcriptome analysis will be considered if results are negative.

Serum testosterone levels will be measured at baseline, during therapy, and after therapy to determine its predictive value for treatment response. In addition, blood samples will also be examined for longitudinal circulating tumour DNA (ctDNA) levels to evaluate whether treatment response and/or failure can be predicted. CtDNA levels will be assessed using an in-house developed and validated hybrid-capture based next-generation sequencing test, as described recently [26, 27].

## Sample size

### Cohort A – CAB-naïve patients

Sample size is calculated based on first primary endpoint, the ORR. Prior data indicate that the response rate among controls (CAB therapy) is 0.417 [4]. If the response rate for experimental subjects is 0.700, we will need to study 37 patients in each arm to be able to reject the null hypothesis that the response rates for experimental and control subjects are equal with probability (power) 0.8. The type I error probability associated with testing our hypothesis is allowed to be 0.1. We will use an uncorrected chi-squared statistic test to evaluate this null hypothesis. Sample size calculation was performed with PS: Power and Sample Size Calculations, version 3.

### Cohort B – CAB-resistant patients

The Simon’s two-stage design, optimal version, will be applied for enrolment in cohort B [28]. The null hypothesis that the true ORR is 5% will be tested against a one-sided alternative, as prior data indicate that the response rate on second-line chemotherapy in patients with SGC is 5% [29]. The combination of the triple therapy will be considered effective and worth of further evaluation if the ORR is at least 20%. In the first phase, nine participants will be accrued and if in at least one of these nine participants a partial or complete response will be observed, fifteen additional participants will be accrued in the second phase of enrolment to a final overall sample size of 24 participants. In case no responses are observed in the first nine patients, cohort B will be closed early for futility. If at least three out of 24 participants confirmed responses are observed, the null hypothesis will be rejected in favour of this triple therapy and this therapy will be considered promising and worthy of further investigation. This design yields a type I error rate of 0.10 and power of 0.8 when the true response rate is 0.20.

### Oversight and monitoring

A Data Safety Monitoring Board (DSMB) is installed to (i) monitor indications of treatment-related harm in both cohort A and B; (ii) review any (serious) adverse events occurring in men and women in both cohort A and B; and (iii) decide continuation of patients recruitment, including the possibility of terminating recruitment for the entire trial or specific cohorts (B) and/or participant subgroups (women). The DSMB is represented by three independent and (inter-)national expert members: an otorhinolaryngologist/head and neck surgeon, a medical oncologist, and a biostatistician. A trial update, including safety measurements, is provided every three months in accordance with the DSMB charter. Furthermore, after completion of the initial phase of cohort B an interim analysis will be provided to the DSMB, according to the results and their recommendations accrual in cohort B will be continued or discontinued. Besides the established DSMB, a clinical research monitor mandated by the Sponsor will monitor the trial according to the Guideline Quality assurance of research involving human subjects for studies with a negligible risk.

### Randomization and data collection

Randomization only occurs for cohort A and will result in the allocation of the control arm and experimental arm in a 1:1 ratio using *Castor EDC*. Randomization to both arms will be performed by variable block randomization (block sizes 2, 4, and 6) and participants will be stratified by sex (male *vs.* female). All data is collected by data managers using the electronic case report form (eCRF) *Castor EDC* to document eligibility, safety and efficacy parameters, compliance to treatment schedules and parameters necessary to evaluate trial endpoints. Patient data will be pseudonymized by allocating a unique patient record ID to all patients. Data will be collected and processed in accordance with the General Data Protection Regulation (EU) 2016/679 and in compliance with national requirements on data protection (*i.e.*, Dutch Personal Data Protection Act). Translational research samples will be stored in the Radboudumc for 25 years in accordance with Dutch national law.

### Statistical methods and data analysis

The primary objective is the best ORR. All evaluable subjects will be included in the analyses. ORRs will be reported as fractions with their corresponding two-sided 95% confidence intervals (CI) per arm. In cohort A, the null hypothesis of equal ORRs in the two arms will be evaluated with a chi-squared test at a significance level 0.10. The difference of the ORRs in the two arms will be reported with a corresponding 90% CI. The (second) primary outcome, the DoR will be reported using descriptive statistics, including median ± CI to draw exploratory clinically relevant conclusions. The data of cohort B will be reported using descriptive statistics (means or medians including CI as applicable).

Patient characteristics will be summarized using means (with standard deviations) or medians (with inter quartile range). Both PFS and OS will be estimated by Kaplan–Meier curves, and for cohort A the difference in survival between arms being evaluated with the log-rank test. CBR and safety will be reported using descriptive statistics (means or medians as applicable) both for cohort A and B. The safety profile will be based on the incidence of SAE’s according to NCI-CTCAE v.5.0. Possible associations between expression of molecular targets and efficacy will be analysed using descriptive statistics, and uni- and multivariable analyses for both cohorts. All patients with a valid baseline and at least one follow-up QoL questionnaire will be included in the analysis per cohort. The baseline questionnaire is considered valid if filled out and dated by the patient before the starting date of trial treatment. Reasons for missing baseline and follow-up questionnaires will be assessed. To evaluate the differences between the treatment groups, with respect to the effect of treatment burden on life-quality during the treatment, the repeated measures of the QLQ-C30 and QLQ-H&N43 will be analysed using mixed effect models. Molecular data will be analysed and reported in accordance with in-house protocols.

## Discussion

A previous clinical trial has shown an ORR of 42% to CAB in palliative setting for patients with R/M AR-positive SDC, and a pre-clinical study showed higher *SRD5A1* expression in patients with a better clinical benefit to CAB therapy in AR-positive R/M SDC. Combining CAB with the SRD5A1-inhibitor dutasteride, might therefore be a more effective treatment than CAB alone in R/M SDC patients, and possibly also in CAB-resistant R/M SDC patients. Our study represents an unmet medical need and focusses on the repurposing of dutasteride to improve therapy response and clinical outcome for patients with R/M SDC having limited other treatment options available, especially when tumours are HER2 negative. By performing the DUCT study, we hope to add value to the clinical practice as most patients with R/M SDC decease due to their disease within a few years. By using a registered low-cost drug, the results of this trial could be promptly transitioned into daily clinical practice.

### Expected limitations

It is important to acknowledge the anticipated limitations inherent to this study. Firstly, our study is a single-institution trial, which may pose challenges in terms of patient recruitment, especially for a rare cancer such as SDC. However, our institution serves as a tertiary referral centre for SGC in the Netherlands. This specialized focus on SGC care not only enhances patient enrolment opportunities but also facilitates research endeavours concerning this uncommon malignancy. Unfortunately, inclusion in cohort A has been closed based on limited accrual (*n* = 2) since April 18, 2024. Secondly, designing and setting up clinical trials for rare diseases presents a unique challenge as it necessitates an acceptable balance between attaining a robust level of scientific evidence and ensuring the practicality of the trial in terms of trial size and duration [30]. Therefore, the Type I error probability for testing our hypothesis is adjusted to 0.1 instead of the conventional 0.05. Lastly, the data related to dutasteride in combination with CAB in castration resistant PCa is limited. Nevertheless, our translational research on SDC provides a compelling rationale suggesting the potential added value of inhibiting SRD5A1 as an adjunct therapy.

### Trial status

The trial is approved on August 11, 2022, with EU-CT number: 2022–500745-24–00. Recruitment started on September 27, 2022. Inclusion in cohort A has been closed since April 2024 due to limited accrual.

## Data Availability

No datasets were generated or analysed during the current study.

## Abbreviations

ACTH: Adrenocorticotropic hormone
ADT: Androgen deprivation therapy
ALT: Alanine aminotransferase
APTT: Activated partial thromboplastin time
AR: Androgen receptor
ARSI: Androgen-receptor signalling inhibitor
ASCO: American Society of Clinical Oncology
AST: Aspartate aminotransferase
CAB: Combined androgen blockade
CBG MEB: Medicines Evaluation Board [Dutch: College ter Beoordeling van Geneesmiddelen]
CBR: Clinical Benefit Rate
CI: Confidence interval
CKD-EPI: Chronic kidney disease epidemiology collaboration
CMO: Committee on Research Involving Human Subjects [Dutch: Commissie Mensgebonden Onderzoek]
CR: Complete response
CRP: C-reactive protein
CT: Computed tomography
ctDNA: Circulating tumour DNA
CTIS: Clinical Trials Information System
DHT: Dihydrotestosterone
DoR: Duration of response
DSMB: Data Safety Monitoring Board
ECOG: Eastern Cooperative Oncology Group
eCRF: Electronic case report form
EORTC: European Organization for Research and Treatment of Cancer
ESMO: European Society for Medical Oncology
HCG: Human chorionic gonadotropin
HER2: Human epidermal growth factor receptor 2
INR: International normalized ratio
LDH: Lactate dehydrogenase
LH: Luteinizing hormone
LHRH: Luteinizing hormone-releasing hormone
MEC-U: Medical research Ethics Committees United
NCI-CTCAE: National Cancer Institute Common Terminology Criteria for Adverse Events
OD: Once daily
ORR: Objective response rate
OS: Overall survival
PCa: Prostate cancer
PD: Progressive disease
PFS: Progression-free survival
PR: Partial response
PSA: Prostate-specific antigen
RECIST: Response evaluation criteria in solid tumours
QoL: Quality of life
R/M: Recurrent and/or metastatic
Radboudumc: Radboud university medical center
RT-qPCR: Reverse transcription-quantitative polymerase chain reaction
SAE: Serious adverse events
SD: Stable disease
SDC: Salivary duct carcinoma
SGC: Salivary gland cancer
SPIRIT: Standard Protocol Items: Recommendations for Interventional Trials
SRD5A1: 5α-reductase 1
TP53: Tumour protein p53
TSH: Thyroid stimulating hormone
VAS: Visual Analogue Scale
WBP: Personal Data Protection Act [Dutch: Wet Bescherming Persoonsgegevens]
WGBO: Medical Treatment Agreement Act [Dutch: Wet Geneeskundige Behandelingsovereenkomst]
WHO: World Health Organization
WMO: Medical Research Involving Human Subjects Act
